# Detailed airflow dynamics and temperature data of axisymmetric and anisothermal jets developing in a room

**DOI:** 10.1016/j.dib.2020.105382

**Published:** 2020-03-05

**Authors:** Teddy Gresse, Lucie Merlier, Frédéric Kuznik

**Affiliations:** Univ Lyon, CNRS, INSA-Lyon, Université Claude Bernard Lyon 1, CETHIL, UMR5008, F-69621 Villeurbanne, France

**Keywords:** Building physics, HVAC, Axisymmetric anisothermal jet, Indoor air temperature, Turbulent flow, CFD validation data

## Abstract

HVAC systems are often used to reach thermal comfort in buildings. It is then necessary to understand the resulting indoor airflow and thermal conditions to optimize the systems. Therefore, this paper presents detailed measurements of axisymmetric and anisothermal jets developing near the ceiling of a thermally controlled room called MINIBAT. Air temperature, velocity and turbulent quantities over five vertical plans on the room were measured using adequate materials such as Pt100 probes and a hot-wire anemometer. These data are to be used for the analysis of indoor air mixing processes and CFD validation. The detailed experimental process and measurements as well as CFD results are exposed in [1].

**Table:** **Specification Table**

Subject	Civil and Structural Engineering.
Specific subject area	Building physics, Ventilation, Fluid dynamics.
Type of data	Tables, figures.
How data were acquired	Potentiometer sensors for positioning measurements, Thermocouples for wall surface temperature measurements, Pt100 probes for air temperature measurements, Hot-wires probes for velocity measurements.
Data format	Raw data collected during measurements are available in .xlsx format.
Parameters for data collection	Three cases of jet temperature are considered: one isothermal case (*Re*=13,360), one hot case (*Re*=21,600) and one cold case (*Re*=11,760).
Description of data collection	The detailed airflow dynamics and temperature measurements of the jet were carried out in a full-scale mechanically ventilated and thermally controlled enclosure called MINIBAT over five vertical sampling plans.
Data source location	CETHIL - INSA de Lyon, France
Data accessibility	Repository name: Mendeley Data Data identification number: 10.17632/hp2cx64vw6.1 Direct URL to data: https://data.mendeley.com/datasets/hp2cx64vw6

## Value of the Data

• The benchmark data can be used for the understanding of the airflow structures and the mixing processes developing in mechanically ventilated and air-conditioned rooms. It also allows to identify the main mechanisms governing the flow and its interactions with the room geometry.
• The data can be useful for engineers working on building physics and HVAC systems, and for researchers for experimental comparison or for validating CFD prediction.
• These data can provide a comparative basis for testing different configurations of air supply systems on rooms. They can further be used for thermal comfort studies e.g. by adding a thermal manikin.

## 1 Data description

**Fig. 1:**
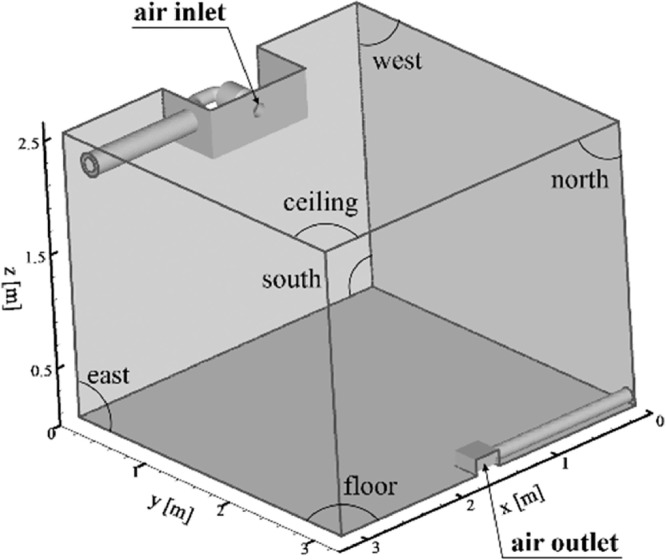
Experimental test cell.

**Fig. 2:**
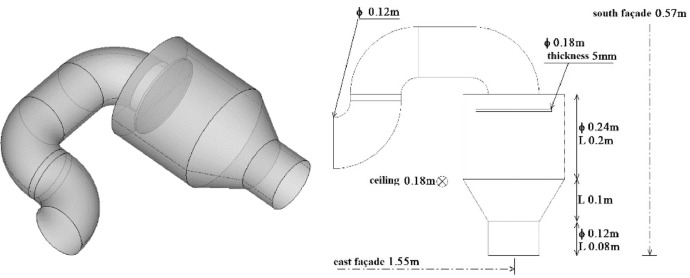
Air supplier system.

**Fig. 3:**
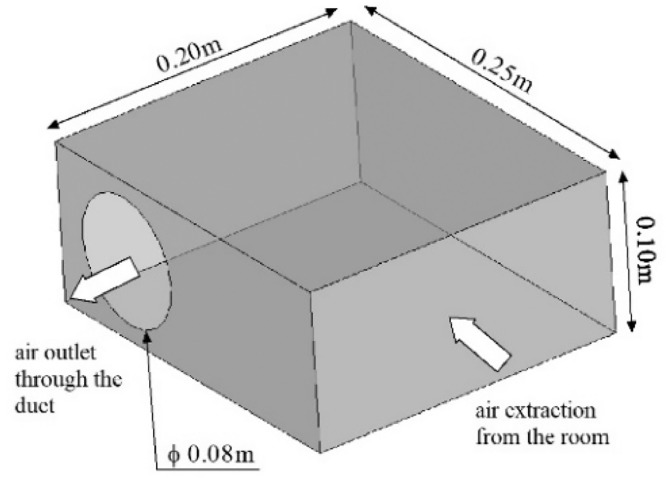
Air extraction system.

The dataset presented in this paper corresponds to the measurement of the indoor air temperature, the mean velocity and the Reynolds stresses induced by an axisymmetric jet developing near the ceiling of a thermally controlled room called MINIBAT. The experimental facility is represented in Fig. 1 and detailed in Fig. 2 and Fig. 3. This data article focuses on three thermal cases from [1]: one isothermal jet, one hot jet and one cold jet. The boundary conditions of the three cases are given in Tables 3 and 4.

The temperature and airflow dynamics measurement data files (reads in .xlsx format) are available in Mendeley Data database under the DOI 10.17632/hp2cx64vw6.1. The database provides separated data files for the three air jet temperature cases organized in folders: ‘Cold case’, ‘Hot case’ and ‘Isothermal case’. Each folder contains five subfolders corresponding to the five vertical sampling plans investigated: ‘Median plan M1’, ‘Transversal plan V1’, ‘Transversal plan T2’, ‘Transversal plan T3’ and ‘Transversal plan T4’. These plans are highlighted in Fig. 6 and the associated sampling parameters are listed in Table 5. For each plan, two files are provided:

• a temperature data file (Temperatures.xlsx) containing the sampling positions and the temperature measurements,
• an airflow dynamics data file (Airflow_dynamics.xlsx) containing the sampling positions, the mean velocity measurements and the Reynolds stress measurements.

Their structures are presented in Tables 1 and 2, respectively.

**Table 1:** Structure of the temperature data files (Temperatures.xlsx).

Location			Temperatures
***x***	*y*	*z*	*T*
(***m***)	(*m*)	(*m*)	(^∘^*C*)

**Table 2:** Structure of the airflow dynamics data files (Airflow_dynamics.xlsx).

Location			Mean velocities			Reynolds stress tensor components					
***x***	*y*	*z*	*Vx*	*Vy*	*Vz*	*txx*	*tyy*	*tzz*	*txy*	*txz*	*tyz*
(***m***)	(*m*)	(*m*)	(*m*/*s*)	(*m*/*s*)	(*m*/*s*)	(*m*^2^/*s*^2^)	(*m*^2^/*s*^2^)	(*m*^2^/*s*^2^)	(*m*^2^/*s*^2^)	(*m*^2^/*s*^2^)	(*m*^2^/*s*^2^)

## 2 Experimental design, materials and methods

### 2.1 Experimental design

The experimental full-scale test cell MINIBAT is represented in Fig. 1. The installation consists of an enclosure whose dimensions are 3.10 m, 3.10 m, 2.50 m according to the coordinate directions (*x*, *y*, *z*). The glazed south façade separates the test cell from a climatic chamber whose temperature is controlled by means of an air-treatment system and kept constant. A thermal guard ensures a uniform temperature of approximately 20 °C on the five other exterior façades.

The axisymmetric jet was generated by an air supplier located on the upper part of the south façade. Its geometry presented in Fig. 2 was specifically designed to obtain the axisymmetric structure of the jet knowing that the air supply duct is positioned on a perpendicular axis compared with the ventilation inlet axis [2]. The air extractor (Fig. 3) is placed on the lower part of the east façade.

The database focuses on three cases for the axisymmetric jet: one isothermal case, one hot case and one cold case. The experiment was carried out under steady-state conditions and the experimental conditions of each selected case are given in Table 3. This table presents the air inlet temperature *T*_0_ [K], the initial Archimede numbers

$$Ar_{0}=\frac{g\beta \left(T_{0}-T_{m}\right)D}{U_{0}^{2}}$$

with *g* [m/s²] the gravitational acceleration, *T_m_* [K] the mean temperature of the non-moving air zone i.e. the zone where the mean velocity is less than 0.05 m/s, β [K^−1^] the air coefficient of expansion, *D* [m] the ventilation inlet diameter and *U*_0_ [m/s] the ventilation inlet velocity, and the initial Reynolds number

$$Re_{0}=\frac{U_{0}D}{\nu }$$

with ν [m²/s] the kinematic viscosity of the fluid.

**Table 3:** Experimental conditions.

Case	*T*_0_ (^∘^*C*)	*Ar*_0_	*Re*_0_	*U*_0_ (*m*/*s*)
Isothermal	21.8	0	13,360	1.67
Hot	30.9	0.0028	21,600	2.70
Cold	12.7	-0.014	11,760	1.47

To have a complete representation of the boundary conditions, Table 4 shows the mean internal surfaces temperatures.

**Table 4:** Mean internal surfaces temperatures (°C).

Case	South	North	East	West	Ceiling	Floor
Isothermal	21.8	21.7	21.7	21.7	21.8	21.7
Hot	24.3	25.0	24.6	24.7	25.5	24.5
Cold	22.6	20.8	21.0	21.0	21.0	20.7

### 2.2 Measurement materials

The inlet and outlet flow rates were controlled by the ventilation system and measured with two flowmeters with a resolution of ±0.5 m^3^/h. The supplied air temperature was measured with Type K thermocouples with a resolution of ±0.4 °C. The six internal surfaces temperatures were measured by the same thermocouples, one face temperature corresponding to the mean value of the nine temperatures measured by the nine thermocouples of each face.

The air temperatures in the room were measured with three miniature Pt100 thermoprobes with a resolution of ±0.2 °C.

**Fig. 4:**
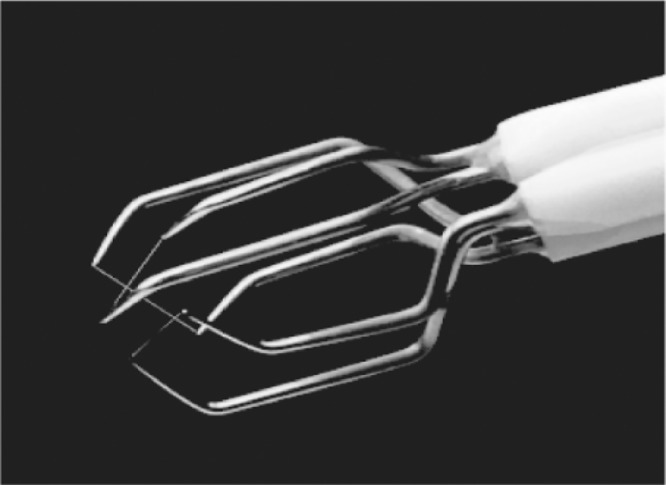
Three-hot-wire probe picture.

The three components of the instantaneous velocity were measured with a three-hot-wire probe DANTEC 55R91 presented in Fig. 4. It is composed of nickel 3 mm long wires with a sensitive part of 1.25 mm. The three wires are inclined at an angle of 54.7° with respect to the probe axis and are in a 3 mm diameter sphere to minimize aerodynamic interference. The probes have been calibrated in-situ along the three directions of the flow, giving an uncertainty on the mean velocity measurement of 0.05 m/s.

Air velocity measurements were corrected using a correlation developed in [1], to avoid errors due to the airflow temperature especially for anisothermal cases. Only velocities with magnitude higher than 0.1 m/s were measured.

### 2.3 Methods

**Fig. 5:**
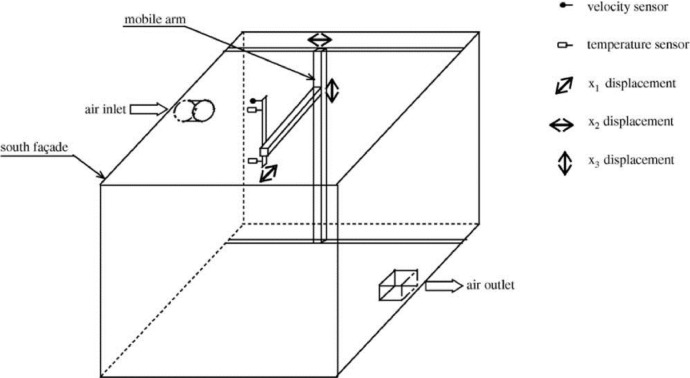
Test cell sensors and mechanical equipment.

The sensors were embedded in a mobile arm to get complete fields of temperature and velocity. Its location in the room was determined by 3 potentiometric resistive sensors set according to the coordinate directions (*x*, *y*, *z*) with a resolution of ±5 mm (Fig. 5).

**Fig. 6:**
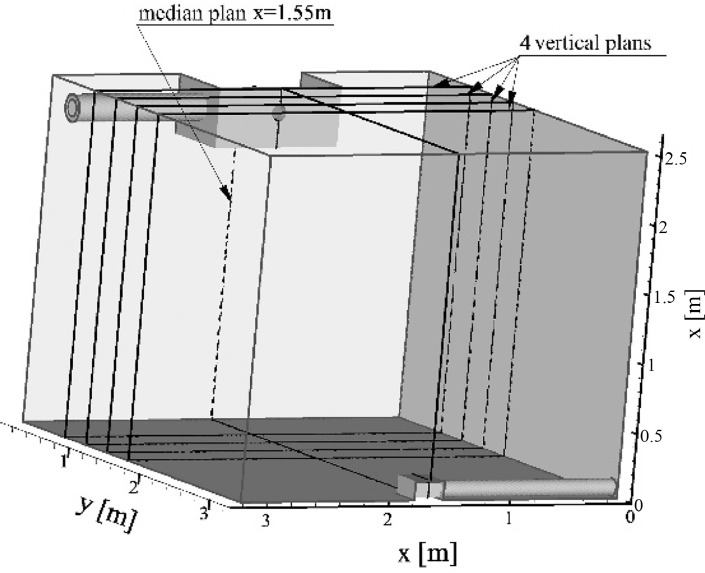
Sampling plans of the test cell.

High resolution measurements were carried out on five vertical plans (Fig. 6) corresponding to:

• A median plan M1 at x = 1.55 m,
• Four transversal plans T1, T2, T3, T4 located at y = 0.60, 0.90, 1.2, 1.5 m respectively.

The amount of sampling varies depending on the air jet temperature case and the plan considered, as mentioned by the Table 5.

**Table 5:** Sampling Parameters.

Plan	Case	Np	Δx	Δy	Δz
			[cm]	[cm]	[cm]
Median	I, C	880	/	5	2
Transversal	I, C	396	2	/	2
Median	F	1760	/	5	2
Transversal	F	792	2	/	2

On these plans, the temperatures were determined by averaging the measures over an acquisition time of 15 s so that an average temperature can be deduced. The velocity measurement rate was 5000 samples per second and the mean velocities in each direction were determined over 150,000 samples, corresponding to an acquisition time of 30 s. These acquisition times were chosen in order to respect the characteristic times of the measured phenomena. The Reynolds stresses were then calculated from the variances of the measured velocity components with a resolution of ±6% of the measured value.

CFD results and validation as well as reporting methods applicable for the benchmark data are further detailed in [1,3] and [4].

## Conflict of Interest

The authors declare that they have no known competing financial interests or personal relationships that could have appeared to influence the work reported in this paper.

## Appendix Supplementary materials

Image, application 1
